# Robot-Assisted Autism Therapy (RAAT). Criteria and Types of Experiments Using Anthropomorphic and Zoomorphic Robots. Review of the Research

**DOI:** 10.3390/s21113720

**Published:** 2021-05-27

**Authors:** Barbara Szymona, Marcin Maciejewski, Robert Karpiński, Kamil Jonak, Elżbieta Radzikowska-Büchner, Konrad Niderla, Anna Prokopiak

**Affiliations:** 1 Sanus Medical Center, Day Treatment Center for Children with Autism, Magnoliowa 2, 20-143 Lublin, Poland; bszymona@gmail.com; 2 Department of Electronics and Information Technology, Faculty of Electrical Engineering and Computer Science, Lublin University of Technology, Nadbystrzycka 36, 20-618 Lublin, Poland; 3 Department of Machine Design and Mechatronics, Faculty of Mechanical Engineering, Lublin University of Technology, Nadbystrzycka 36, 20-618 Lublin, Poland; 4 Chair and I Department of Psychiatry, Psychotherapy, and Early Intervention, Medical University of Lublin, 20-439 Lublin, Poland; 5 Department of Clinical Neuropsychiatry, Medical University of Lublin, 20-439 Lublin, Poland; k.jonak@pollub.pl; 6 Department of Computer Science, Faculty of Electrical Engineering and Computer Science, Lublin University of Technology, 20-618 Lublin, Poland; 7 Department of Plastic, Reconstructive and Maxillary Surgery, Central Clinical Hospital MSWiA, Wołoska 137, 02-507 Warsaw, Poland; eradzikowska@radzikowskaclinic.pl; 8 Dream-Art sp. z o.o., Capital Park, Rejtana 67/5.16, 35-326 Rzeszów, Poland; konrad.niderla@netrix.com.pl; 9 University of Economics and Innovation, Projektowa 4, 20-209 Lublin, Poland; 10 Alpha Medical Center, Warszawska 15, 20-803 Lublin, Poland; anna.prokopiak@poczta.umcs.lublin.pl; 11 Department of Special Psychopedagogy and Special Sociopedagogy, Maria Curie-Sklodowska University in Lublin, Curie-Skłodowskiej 5, 20-031 Lublin, Poland

**Keywords:** autism, robot, therapy, children, communication

## Abstract

Supporting the development of a child with autism is a multi-profile therapeutic work on disturbed areas, especially understanding and linguistic expression used in social communication and development of social contacts. Previous studies show that it is possible to perform some therapy using a robot. This article is a synthesis review of the literature on research with the use of robots in the therapy of children with the diagnosis of early childhood autism. The review includes scientific journals from 2005–2021. Using descriptors: ASD (Autism Spectrum Disorders), Social robots, and Robot-based interventions, an analysis of available research in PubMed, Scopus and Web of Science was done. The results showed that a robot seems to be a great tool that encourages contact and involvement in joint activities. The review of the literature indicates the potential value of the use of robots in the therapy of people with autism as a facilitator in social contacts. Robot-Assisted Autism Therapy (RAAT) can encourage child to talk or do exercises. In the second aspect (prompting during a conversation), a robot encourages eye contact and suggests possible answers, e.g., during free conversation with a peer. In the third aspect (teaching, entertainment), the robot could play with autistic children in games supporting the development of joint attention. These types of games stimulate the development of motor skills and orientation in the body schema. In future work, a validation test would be desirable to check whether children with ASD are able to do the same with a real person by learning distrust and cheating the robot.

## 1. Introduction

Robot-Assisted Autism Therapy (RAAT) has grown in popularity over the past few years [1,2]. Child autism seems to be a particularly promising area of research. The reports from Robot-Assisted Autism Therapy to date prove its effectiveness in various fields, in particular: (1) communication (common attention, imitation, undertaking communication behaviors [3]; (2) recognizing and understanding emotions [4,5]; and (3) developing sensitivity to physical contact [6,7]. Capability to perform therapeutic actions using a remote robot is especially valid during the time of the COVID-19 pandemic, where it is necessary to limit direct interpersonal contact. Robots and advanced robot control systems can also offer additional benefits, like precise gestures and manipulation [8], advanced interaction with the environment [9], facial detection [10], or support of decision making [11].

A child with autism can achieve spontaneous communication, but the learning process requires much more involvement on the part of adults, and often their use of special methods to build dialogue [12,13]. With regard to the recognition and understanding of emotions based on facial expressions, it can be concluded from many papers that there are no significant differences in the perception of people with autism and their properly developing peers. At the same time, research shows that people with autism develop slightly different emotion recognition strategies than neuro-typical people, but they mimic emotional expression as well as those from the control group. Asperger’s Syndrome has already shown that people with autism are characterized by disharmony of emotions. Grandin, in turn, pointed to differences in the subject of emotions [14]. Modern research confirms these arguments—stimuli experienced by people with autism receive a different emotional color than in the case of neuro-typical people. At the same time, research indicates that emotions are an important element of learning and developing for a person with autism, and their development takes place through focusing on relationships [15,16]. Specific sensitivity to physical contact is a characteristic feature for people with autism. It is becoming increasingly common knowledge that the senses of people with autism function differently, including, for example, the sense of touch that is so important in physical contact. Often during medical examinations, parents find out that their child’s sense is correct, e.g., that the child can hear well, but in practice they experience that, e.g., they do not respond to their name, and cover their ears when the vacuum cleaner is working. Children with autism need many different stimulations for the proper development of sensitivity to physical contact. They look for them themselves (e.g., friendly smells, sounds, flavors, or special touch) or receive them from experienced therapists. The development of sensitivity to physical contact is also associated with providing autistic people with proper rest, as such people, often overloaded with excess stimuli, may need special conditions in this regard.

People with autism do not understand many social signals—what gives the feeling of security to most people is usually not enough for them. The search for stability in life can be manifested by a common tendency to put the things of friends over strangers [12,17] or things known over the unknown. One can say that people with autism are desperate to organize the world so that nothing unknown will ever appear in it. They embrace various rules and rituals so that every eventuality can be predicted and that no variables appear in their everyday life. Despite their abilities, some even above average people with autism have low self-esteem and a sense of difference.

## 2. Research Method

This article is a synthesis review of the literature on research with the use of robots in the therapy of children with the diagnosis of early childhood autism. The review includes scientific journals from 2005–2021. Using descriptors: ASD (Autism Spectrum Disorders), Social robots, and Robot-based interventions, an analysis of available research in PubMed, Scopus and Web of Science was done. Criteria adopted to select the papers were: studies about communications, improve social skills in ASD (turn-taking, joint-attention, visual-perspective taking), social functioning and emotional expression, and understanding of emotions. Searching for “ASD + social robots” and “ASD + robot based intervention” yielded 86 and 48 results in PubMed, 337 and 27 results in Scopus, and 246 and 25 results in Web of Science, accordingly.

### 2.1. Learning Social Behavior: Understanding Emotions

Fourteen children (aged 4–8) with ASD diagnosis participated in the research of Marino et al. [15]. These children participated in 10 sessions of Cognitive Behavioral Therapy (CBT) conducted in a group with or without the help of the NAO social work. The research of Marino et al. [15] used the humanoid robot NAO (SoftBank Robotics)—height 58 cm, weight 4.3 kg, 25 degrees of freedom (DOF), touch sensors and audio sensors, actuators (e.g., direct current, or DC motors), and LEDs. The robot acted as a co-therapist, providing the participants with emotional enhancement, and gave hints and tips. In the Marino et al. [15] study, pre- and post-intervention assessments were carried out using TEC (Test of Emotion Comprehension) and ELT (Emotional Lexicon Test). The results of the study showed a statistically significant improvement of contextual recognition of emotions and improvement of understanding of emotions in the group using social robots. Interestingly, in the case of ELT in children with RG (Robot Group), significant improvements occurred not only in recognizing five basic emotions (anger, disgust, fear, happiness, and sadness), but also shame. The intervention had shown that none of the children in the CG (control group) and only two children in the RG were able to recognize and explain what shame is. After a 10-week intervention, all children in RG were able to correctly recognize the emotion of shame measured on the ELT scale, while none of the children from CG were able to correctly recognize this emotion. The authors of the experiment [15] hypothesized to explain in future research: therapy with the help of a robot increased the ability to recognize other emotions as well. It is interesting that in RG all children achieved the highest score in ELT, learning from one to three new emotions, while in CG, no child achieved the highest score and did not learn even one new emotion.

In the study by Valadao et al. [18], high intensity of stereotypical behavior in children with autism was the exclusion criterion. The authors of the study emphasized the importance of its entire first phase (“Self-presentation”). The first phase was innovative in the context of other experiments and also necessary to create a sense of security for the child who works with the robot. The creators of the study thought it important that children who had never seen a robot before were able to get used to it. As children with ASD have difficulty processing large amounts of information and stimuli that can overload them, in turn leading to unnatural behaviors, Valadao et al. [18] decided on complex experiments with a simple design. According to the authors of the study, in the case of the complexity of the experiment, the natural behavior of the child–robot would be difficult to analyze. In the first phase of the experiment, social skills of the subjects were assessed—establishing eye contact—by counting the number of times children looked away from the robot. In the second phase, the assessed social skills related to touch and imitation—the child’s touch of the robot was counted for this purpose. The third phase was the interaction of child–robot–mediator, for this purpose games developing social skills were used. The results obtained in this study were satisfactory.

A work by Japanese specialists—Kumazaki [19]—presents research using two CommU robots. Fifteen children with ASD diagnosis and nineteen healthy boys took part in these studies. Participants were also qualified on the basis of intellectual level assessment: above 80 IQ (intelligence quotient). The study of social functioning was based on the ADOS-2 (Autism Diagnostic Observation Schedule) scenario, where robots improvise three scenes: 1. putting candles on a cake, 2. eating the cake, and 3. cleaning up after the birthday. The study also used a scale assessing the social functioning of patients with the Social Responsiveness Scale.

### 2.2. Non-Verbal Speech (Gestures and Touch)

Interventions with the participation of a robot designed to teach children with ASD various types of gestures have so far been the subject of very little research. The conducted studies showed that people with ASD imitate meaningful gestures better than meaningless gestures [20]. The latter work refers to studies in which Buffington et al. [21] taught children with ASD (aged 4 to 6) nine gestures (e.g., pointing, shaking head) using a structured, behavioral approach. During the training, the therapist presented modeling the correct gesture and verbal response. The child was invited to imitate both: the gesture and the corresponding verbal response. The results showed that children were able to use more appropriate gestures and words after treatment sessions. They were also able to generalize the resulting gesture and verbal responses to new stimuli in a different environment.

So et al. [22,23]—working at the University of Hong Kong—used animation with the help of the NAO robot to introduce learning to understand non-verbal speech (using 20 gestures) in low functioning children with ASD. At least 30% of low functioning school children with ASD do not speak. Teaching these children to recognize and perform gestures would significantly improve their communication skills. In the study presented, the educational program taught children understanding and production of ASD gestures using video modeling (VM) using robot computer animation. Children from 6 to 12 years old with ASD (N = 20; IQ < 70) were taught to recognize 20 gestures made by the robot. The gestures presented by the NAO robot include, for example: the right hand touches the chest (I), both hands clap (delight, “amazing!”), the right hand waves (goodbye), imitates the movement of the bird’s wings, nodding (yes, agreement), clenched fists (anger), and both hands cover the eyes (irritation). Of the children participating in the study, twelve were evaluated by IQ using the Wechsler Intelligence Scale for Children (WISC); their intelligence quotient ranged from 51 to 72 (M = 61.52; SD = 7.87). In the remaining eight, because they were not able to perform subtests in WISC IV, the intelligence quotient was assessed using the Stanford–Binet Scale—their IQ ranged from 49 to 62 (M = 51.18; SD = 4.42). The study also examined the motor and visual abilities of children. In the study of So et al. [22,23], a multi-phase therapeutic intervention program was implemented to teach children with ASD: how to recognize gestures (phase I), then imitate them (phase II), and use them in appropriate social contexts (phase III). The program lasted 12 weeks and each phase 4 weeks. The next phases included: entry tests, four training sessions (2 sessions per week, 30 min each), post-test. At each session, the child was accompanied by a teacher. A small reward (snack, short play) was used to strengthen the positive behavior. In all three phases of the study, significant differences were found between the results of the initial and control tests. Generalization of acquired skills of recognizing and using gestures in the new environment were found in children. These results suggest that VM through robot animation effectively teaches low-functioning children with ASD to recognize and produce gestures in specific social situations.

Touch is an important factor in child development; touch within the origin family is a primary predictor of children’s stable expression of positive feelings [24]. What makes touch “social?” Studies have shown an existing relationship between interpersonal touch and the unmyelinated peripheral afferent fibers (C-touch, or CT fibers). CT fibers answered to gentle, caress-like stroking [25,26]. The relationships between the effects of CT-mediated touch and oxytocin release on pleasant feelings, and social relationships indicates CT fibers as a mediator of oxytocin (OT) [27]. Studies showed that administration of oxytocin correlates inversely with subclinical autism traits [28].

Social touch has an important role in play. Young children who were deprived of touch delivered by parents, or who avoided of touch, did not develop a social play and were at higher risk for sensory processing disorders [29] for example over-sensitivity. Avoidance of social touch in infancy was also a predictor of autism spectrum disorder in preschool children [30].

Animal models of ASD show improper reactions to touch, as well as to light touch with additional developmental influence on social skill [31]. Research has shown that autistic children react to touch in an abnormal way [32,33,34], which is strongly connected to basic clinical symptoms of ASD as well as to biomarkers like white matter pathway excitation [35] and genetic variants increasing functionality of serotonin transporter [34]. Robot-Assisted-Autism Therapy proved to be an important factor in develop of social touch in children with ASD [36,37,38,39].

### 2.3. Anthropomorphic Thinking

Part of the earlier literature on anthropomorphic thinking in TD children shows that children attribute anthropomorphic thinking to inanimate things. For example, according to Kahn et al. [40] most children noticed that the robot is characterized by mental states and that it is a social being that deserves fair treatment and should not be harmed. Social robots were designed in such a way that in many respects they resemble people (movements, speech, appearance, etc.), therefore they were treated by children as a new species of animate or inanimate things, according to the theory of the emergence of a new ontological category [40,41]. Therefore, it would be reasonable to conclude that children can attribute a certain level of anthropomorphic thinking to social robots [5,19]. These studies showed that children with ASD perceived the social robot as more human-like. Children with ASD more often than TD children thought that a robot could grow up and feel pain. The results of the study further confirmed that their perception of human resemblance to robots can affect children’s learning of distrust; children with ASD who attributed a higher level of anthropomorphic thinking to a robot were less likely to learn not to trust it. Despite the clear difficulties in learning complicated social principles, the results of the study by Zhang et al. [4,5] show that children with ASD have better results in contacting the robot than with a real person who performs distrustful tasks.

### 2.4. Complex Social Rules

Twenty Chinese children with ASD and twenty neuro-typical children were qualified for the study [5]. Children were recruited from the community and primary schools from two Chinese cities. Sixteen of the twenty children with ASD (14 boys and 2 girls) in Beijing had a previously confirmed diagnosis through the ADOS. IQ level was determined by the Raven test. The NAO humanoid robot (58 cm tall and 5 kg robot) was used for the research. In the experiment, which lasted 25 min, each child took part in a series of tasks regarding distrust and cheating, training sessions and short interviews on anthropomorphic thinking about robot. The use of a social robot was to teach children complex social rules in this study. Those children who attributed anthropomorphic thinking to the robot showed greater confidence in him. Correlations between anthropomorphic thinking and the level of distrust were carried out for each group separately. The results showed that the negative correlation between anthropomorphic thinking and distrust only existed for the ASD group, r (18) = −0.46, *p* = 0.042, suggesting that those children with ASD who attributed more anthropomorphic thinking to the robot were learning to trust the robot more. In the case of the TD (Typical Development) group, no correlation was found between anthropomorphic thinking and lack of confidence, r (18) = −0.31, *p* = 0.189. Studies have shown that ASD are less likely to learn to distrust and cheat the social robot compared to children with normal development.

Passing on distrust and deception tasks requires an understanding of the mental states manipulating them in others. Hence, it was suspected that TD children participating in this study could learn distrust and cheating by interacting with social robots. However, the findings clearly showed that in both tasks of mistrust and deception, children with ASD could not do as well as TD children. Such results can be explained by the inhibition control found in children with ASD, which can cause difficulties in attributing mental social states.

It is noteworthy that, although children with ASD experienced more difficulties in learning distrust and cheating the robot compared to TD children, they had a similar way of learning as the TD group. Both groups improved their distrust and deception. This result suggests that children with ASD are not completely deprived of all aspects of social learning, they could still, but only to some extent, learn social interaction. However, learning social relations is not as effective as for TD children.

Soleiman et al. [36] used of a parrot-like robot, which is called RoboParrot, as an assistive robot in learning turn-taking skills in children with autism. Authors decided to build a robot for verbal communication. The device is parrot–like because parrots are known as a beautiful animal that can communicate verbally with humans. It was observed that children diagnosed with ASD lack turn-taking skills that are crucial to achieving success in verbal communication. The authors of the study [36] decided to prepare therapy protocols using a parrot-like robot. The robot was used to teach a child turn-taking skills. The therapy was a card game between a child with autism and a therapist or the robot. Card game includes three categories of things: fruits, animals, and body parts. A total of 28 children with ASD participated in the study, from which 19 children did not have turn-taking ability. This study investigated the effectiveness of the robot in therapy. The results showed that the interaction between child and a robot had better effect than between the child and a therapist in most of the turn-taking skills. The study showed that in the situation where the child did not want to cooperate with the therapist, introducing the robot allowed for the session to be completed successfully.

## 3. Relationships and Contact Learning

The literature also describes other examples of using the robot (e.g., Plush) to teach relationships and contact. In recent years, several research teams have undertaken a task of designing a robot that will help children with autism during therapy and daily activities. As a result of these actions, products were created surprisingly different—from a glowing and blinking ball to a miniature boy with an expressive face. It is worth to mention Romibo, a plush blue robot who encourages contact. The robot, looking at the children with eyes on the screen, is controlled by an iPad application that gives the parent or therapist full control over his reactions. In this case, the technology allows you to enter any text on the iPad that the robot will immediately speak or use a database of ready-made scripts. As it can be guessed, the value of Romibo is based on the natural interest that talking and moving stuffed animals arouse in children. The robot seems to be a great tool that encourages contact and involvement in joint activities. Through it you can encourage your child to talk or do exercises. Of course, such contact will not replace neither conversation with the parent nor work with the therapist, it is rather a friendly companion in therapy and games.

The aspect of therapy with autism children discussed here also presents interesting technologies that can give someone a unique voice. Speech synthesizers are commonly used in the communication of people who are not speaking, people with autism form part of their audience. They have only a few standard voices to choose from, so hundreds of people, regardless of age and personality, express their thoughts in the same mechanical voice. The creators of the VocalID platform decided to change it and developed a technology that gives a unique voice to a person [42]. Everyone can give their voice sample via the website. To do this, just spend a few hours recording the sentence list prepared by the creators and send it to the online database. People who need a voice also record their samples—as they are able. Even a single sound allows scientists to read the unique properties of a person’s voice that are used to create a voice specifically for them. However, the parameters are not everything—you need a clear and natural sound. They are provided by recordings donated by volunteers. Advanced technology selects and combines both samples, creating a unique voice that suits the recipient and can be used in alternative communication programs.

Traditional method of teaching “face-to-face instructions from the teacher and parent” is not very attractive and exciting for children with autism [43]. Thus, virtual reality (VR) is one of the most interesting technology for teaching of autistic children. Virtual Reality is more attractive for autistic children and adds a sense of reality [44]. Especially, in-game, the virtual reality can stimulate perception and learning in children. There are a lot of studies that use VR for the developmental training of autistic children with many different activities such as simulation of car driving, simulation of toilet using, and simulation of playing music [44,45,46,47]. The studies focused on developmental training of social skills and behavior skills in ASD [43].

Patsadu et al. [43] proposed a game to develop the cognitive skills for autistic children using virtual reality and estimated satisfaction of the game. The game teaches to develop listening and recognition skills of autistic children in the age of 8-11 years. This game was divided into three parts: developmental training, a game, and reported support for a parent, caregivers, and therapist (Satisfaction Assessment Questionnaire), so that they had information for cognitive development of children. The results showed increased satisfaction of their constructed game in autistic children and in parents.

Taheri et al. [48] evaluated the usefulness of conducting virtual music education programs with automatic assessment system for children with autism. Intervention sessions were conducted for five children with high-functioning autism in age from 6 to 8 years old for 20 weeks, which included a baseline session, a pre-test, training sessions, a post-test, and a follow-up test. Each music education sessions involved teaching different pieces of music according to the child’s cooperation using virtual reality robots and virtual musical instruments.

Among the technological innovations for teaching relationships and contact, there are several solutions that focus on the difficulties in making and maintaining eye contact by people with autism [49]. Some of them, e.g., an application where a smartphone kept in front of the child’s eyes mediates eye contact, raises our doubts. A more interesting solution seems to be the use of glasses for this purpose, which became possible when Google Glass was developed. Glasses that display information in front of the wearer’s eyes, putting them on the viewed image of reality, will someday replace smartphones. The invention is currently used in business and medicine, but one of the companies working with glasses, Brain Power, decided to help children with autism. The Google Glass version intended for them can do a lot at the testing stage. It is equipped with applications that recognize emotions on the faces of the interlocutors, telling you what elements of the image are worth focusing on, encouraging eye contact, and awarding points for looking the interlocutor in the eye. The product can be used both during therapeutic exercises and in natural, everyday contacts with others, which is why it seems as if it has a chance to become something more than a technological curiosity. Much depends on the results of the research on effectiveness and the final price.

The collection of technological innovations supporting the therapy of people with autism also includes movement games that teach how to follow the eyes [44,49]. In Poland, there are games available for children with autism supporting the development of joint attention, based on motion capture technology, i.e., program control by means of movements recorded by a webcam. In addition to social skills, games stimulate the development of high motor skills and orientation in the body schema. Such a game is, for example, Autilius Group Awareness.

## 4. Zoomorphic Robots

### 4.1. Investigation of Stress Biomarkers

In their work, Bharatharaj et al. [6] checked whether therapy through interaction with a robot has an impact on changing the level of stress in children with autism. Seven-week therapy consisted of interaction (playing) of children with the robot—a zoomorphic robot was used for the study: the KiliRo parrot. The results of pioneering studies showed that therapy with a zoomorphic robot reduces the stress level in children with autism. The results of observing the fun of patients with a robot also showed that in some patients playing with a robot improved communication and inhibited impulsive behavior.

### 4.2. Therapy of Emotional Disorders (Anxiety and Impulsive Behavior.)

In Japanese research, Nakadoi [37] used a zoomorphic robot—the Paro seal. The purpose of this research was to check the effectiveness of seal therapy in children with autism. In the psychiatric ward for children and youth, Paro was placed in the corridor, near the door to the nurses’ room. Patients could play with Paro after obtaining permission from employees. It was noted that playing with the robot helped some children improve communication and reduce impulsive behavior and anxiety. However, other patients were afraid of some features of the seal robot, such as large eyes or a slippery nose. These observations lead to the conclusion: before the zoomorphic robot is approved as a therapy tool for patients with ASD, the patient’s approach to the robot should be tested. Undeniably, however, the role of the zoomorphic robot in the therapy of children with ASD is significant.

### 4.3. Developing Fun

Studies by François, Powell, and Dautenhahn [50] were inspired by the approach to therapy through play (Non-Directive Play Therapy). Therapy using a zoomorphic robot was tested in a group of six children with autism at an English school. A zoomorphic robot—a dog—was used for the research. Progress in children was analyzed in three dimensions: naming, playing, and reasoning. The results of the assessment carried out in the case study showed the usefulness of the method to meet the needs and skills of each child. Children who were actively playing socially using the robot gradually experienced a higher level of fun and built more robot-related reasoning.

### 4.4. DreamRobot–System Supporting Therapy of Autistic Children

“DreamRobot–system supporting therapy of autistic children” is a name of a therapeutical-technological project launched in Lublin, Poland [38]. It’s about creating a robot device capable of verbal (speech) and non-verbal (gestures and mimics) communication with children experiencing communication difficulties (in autism). The created device is intended to act as a mediation interface between the therapist and the patient in the course of therapy of developmental disorders. The elimination of direct contact with the doctor/therapist, who is in a way “substituted” with a friendly robot, ready to engage in play, makes it easier for the child to experience the necessary sense of security and generate his or her interest and attention span.

**Figure 1:**
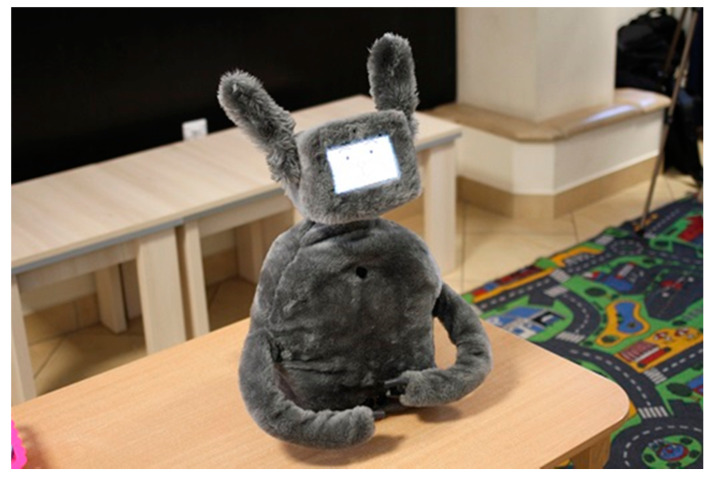
DreamRobot appearance.

The device designed and constructed within the project framework is an anthropomorphic robot resembling a hare or a rabbit (Figure 1). The robot has a number of unique features that make it an innovative product. The key ones include artificial intelligence mechanisms, used not only to recognize users by means of face recognition software, but also to recognize their emotions. In addition, users can be recognized on the basis of speech recording technology. The robot recognizes users’ speech patterns and is also capable of generating language messages with an implemented synthesizer.

**Figure 2:**
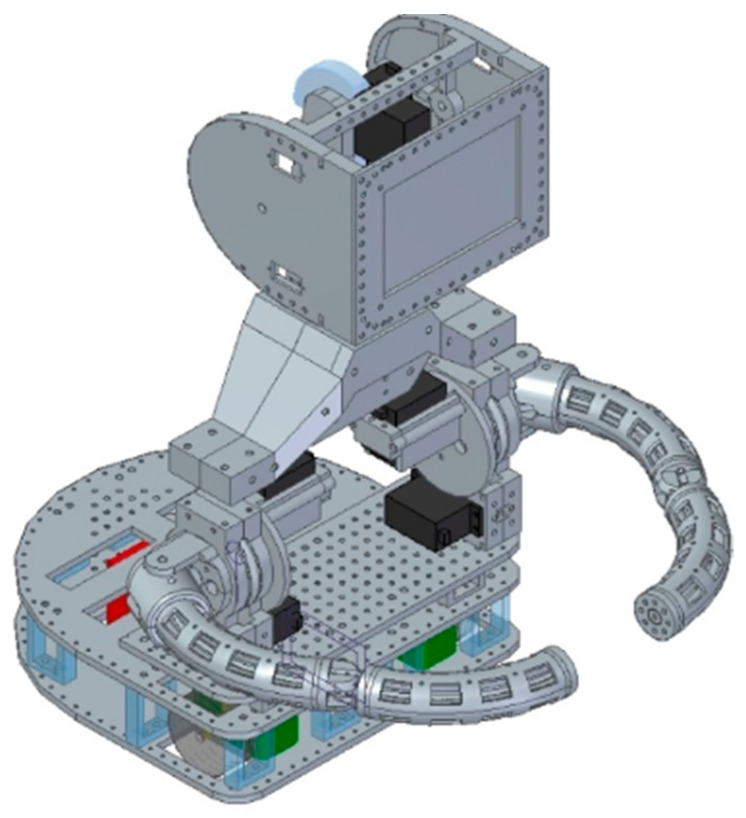
DreamRobot 3D model.

In the creation process many modern technologies were used, such as 3D printing [51], wireless communication [52], and programming languages like C, C++, C#, Java, JavaScript, and Python [38]. SQL and NoSQL data stores are used to collect data obtained during the therapeutic session, and neural networks are used to process data and data mining. The mechanical part was created as a set of 3D printed and laser-cut elements, connected with the use of joints and bearings (Figure 2).

**Figure 3:**
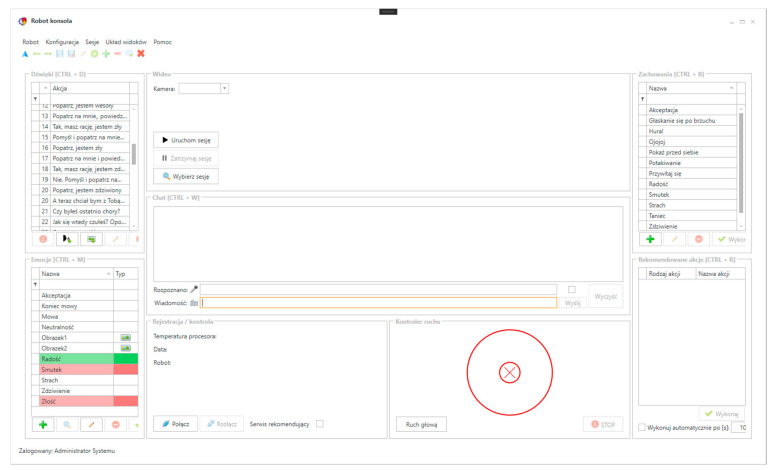
Control panel application, user interface.

The Control panel is the central point of the system, since it brings all parts together into one ecosystem. It is created as a desktop application with a Windows Presentation Foundation (WPF) [53] framework frontend (Figure 3).

**Figure 4:**
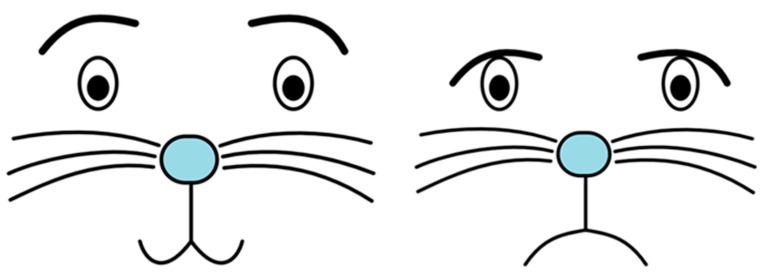
Example of the snout: happy and sad.

The main view of the application is divided into seven parts: Sounds, Emotions, Behaviors, View, Chat, Machine state, and Recommendations. The first three components are dictionaries where own elements can be defined. In the Sounds section user can add items such as frequently used speech sentences, e.g., “Good morning”, “Good bye”, and “How are you?”, or audio files such as jingles or music, etc. In the Emotions section video files like cartoons can be added, or facial expressions of the rabbit can be defined [54]. To define the muzzle a JSON-like format was developed for manipulating facial elements such as eyes, nose, mouth, moustache (Figure 4).

During the session with the patient, the rehabilitator can observe the patient (from the point of view of the device) on the Control panel using the built-in cameras, he/she can also listen to the surroundings using the built-in microphone. In the Chat section the system operator can communicate with the patient. First, the patient’s speech is translated into text and is displayed in the text field. The user can use a predefined item in the Sound section or can write own ad hoc text in the message window. The text is synthesized into speech and played on the robot’s speakers. The user can also use Emotions or Behaviors section item to show a different face on the screen or make a move. The movements can also be performed using the on-screen controller or joystick. The last section described is Recommendations. The machine learning subsystem is a set of many microservices responsible for detecting people [55] and things, emotions, speech and cause-and-effect relationship detection. It also can recognize elements of the environment such as toys and also propose actions such as movement sequences, reactions to the patient, display emotions on the screen, etc. According to the processed data the machine learning gives propositions for the action that should be performed by the DreamRobot.

The robot can perform multiple movements of the selected body parts: the head (3 axes), arms (4 axes), ears (2 axes), and tail (1 axis). As a result, the “animal” may assume a sufficient number of positions to create an impression of the uniqueness of its bodily movements and, consequently, their realistic feel. In addition, the robot, whose height does not exceed 60 cm, moves on wheels hidden in the chassis. The robot can be controlled from the room next to that of the autistic child. This is a great advantage when working with a child on the autism spectrum, for whom a robot working independently is of much greater value than a robot controlled by a therapist visible to the child. The robot should be treated as an intelligent toy, with which the child can talk or play, all during therapeutic sessions.

During the encounters with the robot, children on the autism spectrum showed a noticeably greater interest in the robot than in the therapist. While in the room with the robot, the children asked very personal questions, such as what to do to get the classmates stop teasing them in class, or what to do to get a girlfriend. They were more eager to imitate the movements of the rabbit than those proposed by the therapist. The robot’s own presence significantly increased the motivation of children with autism to perform the tasks proposed by the robot—though indirectly, by the therapist. Limited mimicry of the “muzzle” (in relation to the therapist’s face) was more understandable and predictable, and thus being in the zone of proximal development of children on the autism spectrum, allowed them to learn to understand various emotions expressed by people. The robot became a bridge to their better understanding of the world of emotions of neurotypical people.

The authors of the paper have experience with robots in the treatment of children with ASD. A zoomorphic robot DreamRobot was built and first presented at a conference in 2019. Clinical trials with DreamRobot were conducted in 2019 and 2020 on 19 children with autism and they took place at two centers for rehabilitation of autistic children. The overall response of the children and staff is very positive. The robot project is under continuous development.

## 5. Future Research Directions

The latest research shows that it is still challenging to diagnose children on the autism spectrum using robots. The work of diagnosticians and personnel is difficult and time consuming. Particular behavioral patterns of people on the autism spectrum could be used to identify risk factors by means of robotic interaction [56].

Parents of children on the autism spectrum sometimes lack access to a specialist. In these situations, it might be possible for a robot to perform certain tasks and achieve goals prepared by a specialist located remotely. This requires further study and work towards more advanced robots and tools to assess their functionality in autistic children [57,58].

Initiating of social interaction is one of the main deficits among those from the autism spectrum. A study of the subject was conducted by De Korte, Berk-Smeekens, and Dongen-Boomsma [59] on a group of 44 children aged 3–8 with autism disorders. According to the results, the number of social initiatives originating from the child increased during sessions where a supporting robot was used. The children would follow the therapy protocol better when a robot was used (average 85.5%), showed positive effects after the therapy sessions (positive 86.6%), and expressed sympathy and positive feelings towards the robot (high in 79.4%). Highest scores were mostly present in children of school age (H(1) = 7.91, *p* = 0.005). They usually mentioned positive attitude towards robotic motions, speech, and game scenarios. Parental opinions about the use of robotic support in therapy were mostly positive (average 84.8%), while lower scores usually were given due to lack of elasticity in robotic behavior.

Research by Ramírez-Duque, Aycardi, Villa et al. [60] was conducted in a group of 20 children on the autism spectrum. During the study they were asked to participate in 14 lessons which featured the use of a robot. The lessons were conducted using applied behavior analysis (ABA). The analysis showed that sensory rewards delivered by the robot resulted in more positive reactions than spoken praise from people. The results were compared with a control group that played ball together. Children from this group expressed more prosocial behaviors that those participating in lessons using the robot. Despite this, the therapy sessions that included the presence of the robot were regarded higher by both children and parents. The role of robots in therapy of children on the autism spectrum requires more analyses.

A research based on the ADOS [61] tool included over 3000 therapeutical sessions and 300 h of therapy for 61 children diagnosed with autism spectrum disorders. Therapy was conducted using ABA. Half of the group participated in lessons using the robot, while the other half in lessons with the therapist only. In the research, Robot Enhanced Therapy (RET) was presented as potentially cost effective, although wide scale clinical testing is still lacking [62,63,64]. Data collected by the authors can be used in further study on the subject using machine learning or artificial intelligence. Results can be helpful in further clinical trials or be a basis for new therapeutical methods, especially for the people on the autism spectrum [65]. Modern models of robots introduce new possibilities in development of personalized autonomic systems targeted at people with non-typical cognitive, affective, social, and emotional needs [66,67]. Some of the latest works point out the role of individualized tactile perception of the robot [68,69].

Most recent studies indicate that use of Virtual Reality (VR) technology may be beneficial. Autistic children are one of the groups of potential users, as they require support towards social interactions. This technology could help them train social skills by virtual world simulation. This would allow for therapy without need to buy the robot itself, giving the option of cheaper therapy [45]. Use of the robot can be beneficial in schools, when it can be used to support teleconsultation and video conferences [70].

Promising results in improving skills of visual perspective-taking (VPT) and theory of mind (ToM) were achieved using new methodology working with children with autism spectrum disorder (ASD) using a humanoid robot, Kaspar [71]. VPT is an ability to see and interpret the world from a different person perspective using social and spatial information. Children with ASD often find it hard to understand, that other people may have different perspective, opinion or point of view than their own, which is a basic aspect of VPT as well as ToM. Games designed during the study were the first attempt at testing whether these skills can be improved in children by interacting with a humanoid robot in a series of trials. The results suggest that children with ASD can actually benefit from this approach towards robot assisted therapy.

## 6. Conclusions

Based on the literature review, the most common inclusion criteria can be identified [4,5,61]:

(1) Age between 4 and 11 years old;
(2) Clinical diagnosis of childhood autism based on the Autism Diagnostic Observation Schedule (ADOS-2) study and International Classification of Diseases (ICD-10) criteria;
(3) Assessment of the severity of clinical symptoms in ADOS-2 from mild (level 1) to moderate, both in social communication and interaction by experienced specialists in the research team (psychiatrist, special pedagogue, and clinical psychologist);
(4) Sufficient verbal level and intelligence quotient above 70;
(5) No current problems with aggressive behavior or increased opposition-rebellious disorders;
(6) Lack of auditory, visual or physical disability that would prevent participation in the study;
(7) Not using psychiatric drugs;
(8) The child is not subjected to any other intervention directly related to emotions or social skills throughout the study;
(9) Lack of neurological treatment and neurological diseases.

The exception is the work of So et al. [22,23], where research on learning to understand non-verbal speech (gesticulation) not speaking to children with a diagnosis of autism and accompanying mental retardation (intelligence quotient below 70) was qualified. Criteria 5, 7, and 8 were not considered in the study of psychiatric ward patients using the Paro seal robot.

Children with ASD can approach the robot as if it were a new species, especially at the beginning of the learning process. When they become familiar with the robot, interacting with it, they gradually develop anthropomorphic thinking about the robot and perceive it more and more as a human being or the essence of a new species than a toy or technical device. As mentioned earlier, this development of anthropomorphic thinking can hamper learning of distrust in children with ASD. A summary of the robotic solutions presented in this paper is shown in Table 1.

This review of the literature on the subject orientates in specific therapeutic predispositions of robots, in relation to learning the principles and social skills of children with ASD. The area of the potential use of robots is to shape distrust, understanding gestures and emotions, and imitation. The results of previous experiments also shed light on future research that should address social issues. In this context, the question should be asked: can learning from robots be generalized to a universal case (e.g., does distrust or cheating of a robot contribute to the effect of distrust or cheating of a real person?). In future work, a validation test would be desirable to check whether children with ASD are able to do the same with a real person by learning distrust and cheating the robot.

Supporting the development of a child with autism is a multi-profile therapeutic work on disturbed areas, especially understanding and linguistic expression used in social communication, development of mutual social contacts and functional or symbolic play. Previous studies hopefully allow one to think about therapy using a robot. Autistic disorders are very diverse in nature, they do not form a uniform picture as to the symptomatology and depth of the disorder. It seems that in particular a robot could: (1) encourage contact, (2) suggest during a conversation, (3) teach, and entertain.

In the first aspect, the value of a robot is based on the natural interest that the speaker and the robot induce in children. The robot seems to be a great tool here that encourages contact and involvement in joint activities. Through it, one can encourage the child to talk or do exercises. In the second aspect (prompting during a conversation), a robot that recognizes the emotions on the faces of the interlocutors, suggesting what elements of the image are worth focusing on, encourages eye contact and suggests possible answers, e.g., during free conversation with a peer. In the third aspect (teach, entertain) the robot could play with autistic children in games supporting the development of joint attention, based on motion capture technology, i.e., program control by means of movements recorded by a webcam. These types of games, apart from social skills, stimulate the development of motor skills and orientation in the body schema.

To sum up, it should be stated that the research indicates the potential values of the use of robots in the therapy of people with autism as a facilitator in social contacts and as such they are worth further research.

## Figures and Tables

**Table 1:** Summary of robotic solutions for Robot-Assisted Autism Therapy.

Author	Type of Robot	RAAT Study Area	Results
Kumazaki et al. (2019) [19]	2 Humanoid robots CommU	Communication and Social Skills	Improvement of social functioning based on the ADOS-2 scenario.
Wood et al. (2019) [39]	Humanoid robot Kaspar	Communication and Social Skills	Improvement of social skills.
Zhang et al. (2019) [5]	Humanoid robot NAO	Communication and Social Skills	Children with ASD have better results in contacting with the robot then with a real person who performs distrustful tasks.
Marino et al. (2020) [15]	Humanoid robot NAO	Recognizing and Understanding Emotions	Improvement of contextual recognition of emotions and improvement of understanding of emotions in the group using social robots.
Niderla and Maciejewski (2021) [38]	Zoomorphic DreamRobot	Developing Sensitivity to physical contact, Recognizing and Understanding Emotions	After 6 weeks therapeutic sessions learning and recognizing emotions in face of rabbit, developing of spontaneous and imaginative play; developing social touch.
Soleiman (2021) [36]	Zoomorphic Robot RoboParrot	Verbal Communication and Social Skills	Improvement in turn-taking skills.
Nakadoi et al. (2017) [37]	Zoomorphic Robot Paro seal	Social touch	Reduce impulsive behavior and anxiety.
Buffington et al. (1998) [21]	Humanoid robot NAO	Non-verbal speech	Correct gesture and verbal response, teaching children nine gestures (pointing, shaking head).
So et al. (2016) [22]	Humanoid robot NAO	Non-verbal speech	Learning to understand 20 gestures in low functioning children with ASD.
Valadao et al. (2016) [18]	Humanoid robot MARIA	play games	Game developing social skills.
Shahab et al. (2021) [45]	social Virtual Reality Robot	Education	Educational support in children with high-functioning autism.
Patsadu (2019) [43] Liu et al. (2018) [72] Feng (2017) [49] Redwood et al. (2017) [51]	VR game	Education	Assist with education in many areas: social communication, behavioral skills, simulated music playing or driving.

## Data Availability

The data presented in this study are available on request from the corresponding authors.
